# Cloning, expression, purification and characterization of chitin deacetylase extremozyme from halophilic *Bacillus aryabhattai* B8W22

**DOI:** 10.1007/s13205-021-03073-3

**Published:** 2021-12-01

**Authors:** Goutam Mohan Pawaskar, Keyur Raval, Prathibha Rohit, Revathi P. Shenoy, Ritu Raval

**Affiliations:** 1 Department of Biotechnology, Manipal Institute of Technology, Manipal Academy of Higher Education, Manipal, 576104 India; 2 Department of Chemical Engineering, National Institute of Technology Karnataka, Surathkal, 575025 India; 3 ICAR-Central Marine and Fisheries Research Institute, Mangalore, 575001 India; 4 Department of Biochemistry, Kasturba Medical College, Manipal Academy of Higher Education, Manipal, 576104 India

**Keywords:** Receptor plate assay, *Bacillus aryabhattai* B8W22, Chitin deacetylase extremozyme, Lactose induction, Halotolerant, Thermostable

## Abstract

Chitin deacetylase (CDA) (EC 3.5.1.41) is a hydrolytic enzyme that belongs to carbohydrate esterase family 4 as per the CAZY database. The CDA enzyme deacetylates chitin into chitosan. As the marine ecosystem is a rich source of chitin, it would also hold the unexplored extremophiles. In this study, an organism was isolated from 40 m sea sediment under halophilic condition and identified as *Bacillus aryabhattai* B8W22 by 16S rRNA sequencing. The CDA gene from the isolate was cloned and overexpressed in *E. coli* Rosetta pLysS and purified using a Ni–NTA affinity chromatography. The enzyme was found active on both ethylene glycol chitin (EGC) and chitooligosaccharides (COS). The enzyme characterization study revealed, maximum enzyme velocity at one hour, optimum pH at 7 with 50 mM Tris–HCl buffer, optimum reaction temperature of 30 ºC in standard assay conditions. The co-factor screening affirmed enhancement in the enzyme activity by 142.43 ± 7.13% and 146.88 ± 4.09% with substrate EGC and COS, respectively, in the presence of 2 mM Mg^2+^. This activity was decreased with the inclusion of EDTA and acetate in the assay solutions. The enzyme was found to be halotolerant; the relative activity increased to 116.98 ± 3.87% and 118.70 ± 0.98% with EGC and COS as substrates in the presence of 1 M NaCl. The enzyme also demonstrated thermo-stability, retaining 87.27 ± 2.85% and 94.08 ± 0.92% activity with substrate EGC and COS, respectively, upon treatment at 50 ºC for 24 h. The kinetic parameters *K*_m_, *V*_max_, and *K*_cat_ were 3.06E−05 µg mL^−1^, 3.06E + 01 µM mg^−1^ min^−1^ and 3.27E + 04 s^−1^, respectively, with EGC as the substrate and 7.14E−07 µg mL^−1^, 7.14E + 01 µM mg^−1^ min^−1^ and 1.40E + 06 s^−1^, respectively, with COS as the substrate. The enzyme was found to be following Michaelis–Menten kinetics with both the polymeric and oligomeric substrates. In recent years, enzymatic conversion of chitosan is gaining importance due to its known pattern of deacetylation and reproducibility. Thus, this *Ba*CDA extremozyme could be used for industrial production of chitosan polymer as well as chitosan oligosaccharides for biomedical application.

**Supplementary Information:**

The online version contains supplementary material available at 10.1007/s13205-021-03073-3.

## Introduction

Chitin is the second-most abundant biopolymer followed by cellulose. The application of chitin is limited due to its crystalline structure and insoluble property. Chitosan is the deacetylated form of chitin, soluble in slightly acidic conditions. The cost-effective commercial synthesis of chitosan is done by chemical deacetylation (Mathew et al. 2021). The chemical conversion compromises the quality of chitosan concerning reproducibility in physiochemical properties like degree of polymerization (DP), degree of acetylation (DA) and the pattern of acetylation (PA) (Cord-Landwehr et al. 2020; Wattjes et al. 2020). The volumes of effluents generated in the chemical process also add to an environmental load of pollutants. Over the years, several studies have shown the medical application of chitosan, but the use of chemically converted chitosan is limited due to its physicochemical properties. Therefore, enzymatic deacetylation of chitin is gaining attention for medical applications (Hu et al. 2021).

Chitin deacetylase (CDA) (EC 3.5.1.41) is a hydrolytic enzyme that belongs to carbohydrate esterase family 4 (CE-4 s) as per the CAZY database (Carbohydrate Active Enzymes database), URL (http://www.cazy.org) (Lombard et al. 2014; Andreou et al. 2018). CDA hydrolyses the acetamido group in chitin to form chitosan, releasing acetic acid as a by-product (Pawaskar et al. 2019). Structurally, the CE-4 family enzymes share the NodB homology domain or polysaccharide deacetylase catalytic domain as a conserved region. In some studies, the chitin-binding domain is also reported (Grifoll-Romero et al. 2018). The first CDA discovered from *Mucor rouxii* fungus and *Colletotrichum lindemuthianum* is the most-studied organism for its CDA activity (Ghormade et al. 2010). Other fungi like *Aspergillus flavus*, *Mortierella* sp., *Absidia coerulea*, *Rhizopus circinans*, *Penicillium oxilium*, and bacteria like *Vibrio alginolyticus*, *Bacillus* sp., *Alphaproteobacteria* have been recently reported for their CDA activity (Kaczmarek et al. 2019).

As the commercial source of chitin is marine waste, the interest in isolating CDA-producing micro-organisms from aquatic sources has increased (Mathivanan et al. 2021). In the previous study, we reported a rapid and sensitive agar-based CDA screening method (Pawaskar et al. 2019). In the present finding, we report the isolation of halophilic bacteria from sea sediment at a depth of 40 m for CDA activity using this novel receptor-based screening method (Pawaskar et al. 2019). The bacteria having maximum CDA activity was later identified as *Bacillus aryabhattai* B8W22. The CDA gene from *B. aryabhattai* B8W22 (*Ba*CDA) was cloned and heterologously expressed in *E. coli* Rosetta pLysS cells. The *Ba*CDA was purified using Ni–NTA affinity chromatography and characterized with ethylene glycol chitin (EGC) and chitin oligosaccharide (COS) as substrates. Enzyme kinetic parameters were also determined.

## Materials and methods

### Chemicals and reagents

Ethylene glycol chitosan (EGCS), Chitin oligosaccharide (COS), Empty polypropylene SPE Tube with PE frits, and Primers were purchased from Sigma Aldrich, India. Q5 Hi-fidelity Taq DNA polymerase, restriction enzymes, DNA ligase, and Monarch® Plasmid Miniprep Kit were purchased from New England Biolabs (NEB) India. PCR product/gel extraction kit was purchased from Promega India. A glucose assay kit was purchased from Agappe diagnosis Pvt. Ltd., India. Ni–NTA agarose beads were purchased from QIAGEN India Pvt. Ltd., India. Dialysis membrane was purchased from Thermo-fisher scientific India, Amicon® Ultra-15 Centrifugal Filter Unit was purchased from Merck-Millipore, India. Acetate calorimetric assay kit was purchased from Megazyme (Ireland). All other chemicals and reagents were of analytical grade.

### Plasmid, bacterial strains, and culture media

pET22b (+) DNA—Novagen was purchased from Merck-Millipore, India, and stored at − 20 ºC. *E. coli* DH5α and *E. coli* Rosetta pLysS—Novagen cells were purchased from Merck-Millipore, India, and maintained in Luria–Bertani (LB) broth containing 25% glycerol at − 80 ºC. *Bacillus aryabhattai* B8W22 was isolated in our lab and maintained in Nutrient Broth (NB) containing 25% glycerol at − 80 ºC.

### Sample collection, isolation, and screening of CDA-producing bacteria

An Arabian Sea sediment sample was collected from a depth of 40 m using Ekman dredge with the coordinates as 12° 48′ N and 74° 40′ E. The sample was transported in a sterile container to the laboratory immediately and stored in a cold room till further use (Anas et al. 2016). A 100 µL of serially diluted sample solutions was inoculated by spread plate method on the colloidal chitin plate containing NaNO_3_—2 g L^−1^, K_2_HPO_4_ — 1 g L^−1^, KH_2_PO_4_—1 g L^−1^, MgSO_4_—0.5 g L^−1^, colloidal chitin—1% (w/v) and agar—2.5% (w/v) at final concentration, dissolved in synthetic seawater (Anas et al. 2016; Pawaskar et al. 2019). The plate was cultured for 7 days at 37 °C. The isolated bacteria were sub-cultured multiple times on a fresh colloidal chitin plate to get a purified single colony. The purified single colony was spot inoculated on a receptor-based screening plate for CDA activity.

### Crude enzyme activity of the isolates

The positive isolates from the receptor plates were inoculated in liquid broth containing NaNO_3_—2 g L^−1^, K_2_HPO_4_—1 g L^−1^, KH_2_PO_4_—1 g L^−1^, MgSO_4_—0.5 g L^−1^, colloidal chitin—1% (w/v) at final concentration, dissolved in synthetic seawater. After culturing the isolate for 48 h at 37 ºC with 180 rpm agitation, the supernatant was collected as crude extract followed by centrifugation at 5405*g* for 10 min at 4 ºC. The extracellular CDA activity of the crude extract was determined by acetate assay kit as per the manufacturer’s instructions. Ethylene glycol chitin (EGC) was used as a substrate for the reaction (Araki and Ito 1988). One unit of the enzyme is defined as the activity which released 1 μmol of acetate from the substrate per microgram of enzyme per minute. All the enzyme assays were carried out in triplicates and corrected for background from control reactions, one without enzyme and another without substrate (Raval et al. 2013, 2017).

### Identification of bacteria having maximum CDA activity

The isolate yielding maximum CDA activity was identified by 16S rRNA gene analysis. 16S rRNA gene was amplified using 27F: AGA GTT TGA TCM TGG CTC AG and 1492R: CGG TTA CCT TGT TAC GAC TT as forward and reverse primer, respectively. By Sanger sequencing, the 16S rRNA gene was analysed and compared with the gene database using the BlastN algorithm to determine the relative position of strain in phylogeny. Multiple sequence alignment of the top 10 blast hit was done using the MUSCLE algorithm and a phylogenetic tree was constructed using MegaX software. The isolate was identified by the closest neighbouring strain in the phylogenetic tree (Kim and Chun 2014; Kumar et al. 2018).

### Gene identification, annotation, and cloning

The homology-based comparative approach was used to predict the CDA gene sequence. In brief, an already reported putative CDA gene sequence from highly related species was retrieved from the NCBI gene database. These sequences were used to search the whole genome sequence of the isolate by the BLAST server. The homologous region was annotated as the CDA gene in the NCBI gene data bank.

A set of primers (forward primer: 5′-GCCGCCGCATATGATGAATATGTTTTATAC-3′ and reverse primer: 5′-GTATCTCGAGGCGGATATCTTTTACTTG-3′) was designed using Benchling web tool (https://benchling.com). The primer was designed to include *Nde*I and *Xho*I restriction sites in the sequence. A virtual clone was constructed using SnapGene software (https://www.snapgene.com). The designed primer was synthesized and purchased by Sigma Aldrich, India.

The *Bacillus aryabhattai* B8W22 gDNA was isolated using the phenol/chloroform extraction method for the amplification of the CDA gene (Andreou 2013). The optimized PCR condition included 30 cycles comprising denaturation at 94 °C for 10 s, annealing at 58 °C for 30 s, extension at 72 °C for 60 s. The amplified gene product was purified as per the user’s manual. The amplified *Ba*CDA gene and pET22b (+) vector were digested with *Nde*I and *Xho*I restriction enzymes and purified as per the user’s manual. The digested vector and insert were ligated in the ratio 3:1 using a T4 DNA ligase at 16 °C for 16 h. The ligated product was transformed into *E. coli* DH5-α competent cells and plated on LB agar plate containing ampicillin antibiotic (100 µg mL). The successful cloning in the transformed cells was verified by colony PCR and restriction digestion of the isolated plasmid. The final confirmation of the insert was achieved by PCR using universal T7 promoter and T7 terminator primers. Homology modelling with PDB ID 1W17, 2C1G, 2CC0, 2IW0, and 2Y8U was undertaken for the translated cloned sequence. The conserved region in *Ba*CDA was determined by ESPript 3.0 (http://espript.ibcp.fr) online tool (Robert and Gouet 2014).

### Expression and purification of *Ba*CDA

The cloned plasmid was transformed into *E. coli* Rosetta pLysS competent cells and plated on LB chloramphenicol (35 µg mL^−1^), ampicillin (100 µg mL^−1^) agar plate. The overexpression of recombinant *Ba*CDA was optimized by one variable at a time method. The variables considered were temperature, induction OD_600_, IPTG concentration, induction time and induction media.

The overexpressed cells were centrifuged at 5405*g* for 10 min at 4 ºC for harvesting. The lysate was prepared by adding 5 mL of lysis buffer (50 mM Tris–HCl buffer pH 8, 300 mM NaCl, 5 mM imidazole) to each gram of pellet. The lysis included 20 cycles of sonication with 5 s on and 10 s off phase. The amplitude maintained for lysis was 60%. All the experiments were performed on ice. The lysate was collected after centrifugation at 5405*g* for 10 min at 4 ºC. The lysate was then subjected to purification by the Ni–NTA affinity chromatography. Briefly, the column was washed with Milli-Q water and equilibrated with lysis buffer. The *Ba*CDA in the lysate was allowed to bind to the Ni–NTA column by passing through gravity flow. The column was washed with wash buffer (50 mM Tris–HCl buffer pH 8, 300 mM NaCl, 50 mM imidazole, 10% glycerol) and the bound enzyme was eluted with elution buffer (50 mM Tris–HCl buffer pH 8, 300 mM NaCl, X imidazole) with an imidazole concentration of 250 mM and 500 mM. The purified elution was pooled and dialyzed at 4 ºC against 50 mM Tris–HCl buffer pH 8 using a 10 kDa cut-off dialysis membrane. *Ba*CDA was later concentrated using a 10 kDa Amicon® Ultra-15 Centrifugal Filter Unit by centrifuging at 2111*g* for 30 min at 4 ºC. The final yield and purification fold were calculated for the purified *Ba*CDA.

### Expression scale-up and investigation of lactose induction point

The expression was scaled up to 1 L in a shake flask under optimized conditions. The yield was found by measuring the biomass (wet pellet weight). To understand the role of lactose in induction and repression by glucose, sampling at every four hours of the interval was done and the assay parameters were analysed.

### Enzyme characterization

The enzyme was characterized by determining the enzyme activity using EGC and COS as substrates. The initial enzyme activity experiments were carried out in 50 mM Tris–HCl (pH 7) buffer at 37 ºC. The enzyme velocity was determined at 30–360 min time intervals. The suitable pH and buffer conditions were studied by testing enzyme activity in different pH and buffer conditions. A pH range was of 4–10 using the following buffers: 50 mM citrate buffer (4–6), 50 mM bis–tris buffer (6–7), 50 mM phosphate buffer (6–8), 50 mM Tris–HCl buffer (7–8), 50 mM boric acid buffer (8–9) and 50 mM carbonate–bicarbonate buffer (9–10). The optimum reaction temperature was determined by assaying the *Ba*CDA activity at various temperatures (20–60 °C).

The effects of monovalent metal ions (K^+^, Na^+^), divalent metal ions (Ca^2+^, Co^2+^, Zn^2+^, Mn^2+^, Mg^2+^, Fe^2+^, Ni^2+^,) and EDTA on the *Ba*CDA activity were estimated by pre-incubating the *Ba*CDA in the 50 mM Tris–HCl buffer (pH 7) with a metal ion at a concentration of 1 mM. After one hour of reaction, residual activity was measured under standard assay conditions. The activity determined in the absence of metal ions was recorded as 100%. The acetate inhibition on *Ba*CDA activity was also determined by assaying the enzyme activity in presence of acetate with concentrations ranging from 0.1 to 10 mM under standard assay conditions.

The salt tolerance and thermal stability of *Ba*CDA were determined. The enzyme activity using acetate assay kit at standard assay condition was carried out in presence of 0 mM to 2 M NaCl. The enzyme was incubated for 24 h at 4 ºC, 20 ºC, 30 ºC, 40 ºC, 50 ºC, 60 ºC, 70 ºC, and 80 ºC in optimum buffer, metal ion, and salt condition. After the incubation time, the enzyme activity was assayed to know the thermal stability of the *Ba*CDA.

### Enzyme kinetics

The kinetic parameters of purified *Ba*CDA were determined by enzyme activity under optimized conditions using an acetate assay kit. The Michaelis–Menten kinetic constants (*K*_m_ and *V*_max_) were calculated by plotting the Lineweaver–Burk graph (Lineweaver and Burk 1934). The substrate EGC at concentrations ranging from 1.00 to 5.00E−7 µg mL^−1^ and COS at a concentration ranging from 1.00 to 1.00E−8 µg mL^−1^ were used. The enzyme specificity towards the substrate chain length was determined using two types of substrates.

## Results

### Isolation, screening, and identification

**Fig. 1:**
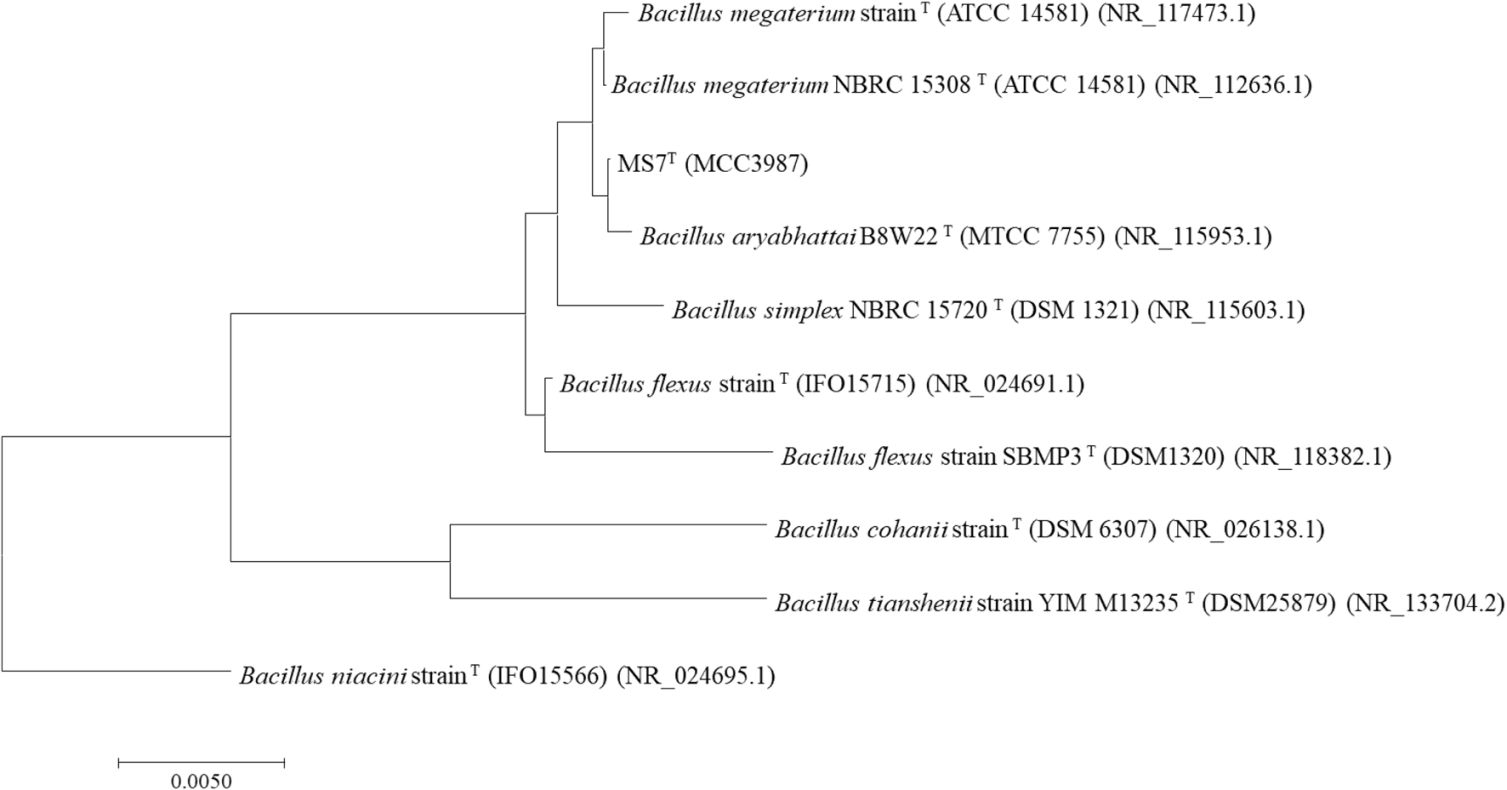
The Phylogenetic analysis. The phylogenetic tree showing the isolate MS7 was highly homologous with *B. aryabhattai* B8W22 with a 99% match. The *Bacillus niacini* was taken as an out-group. The phylogenetic tree was constructed by a maximum parsimony algorithm. The names of the culture collection centres of the type isolates compared for the phylogenetic tree are American Type Culture Collection (ATCC), Leibniz-Institut DSMZ-Deutsche Sammlung von Mikroorganismen und Zellkulturen GmbH (DSM), Institute for Fermentation, Osaka (IFO), Microbial Type Culture Collection (MTCC)

Fifteen morphologically different colonies were observed on the colloidal chitin agar plate after 7 days of incubation. Out of fifteen, four isolates were tested positive for CDA activity, showing fluorescence around the colony on receptor-based screening plate on the fourth day of incubation (Fig. S1). The crude CDA activity of the four isolates was further quantified using acetate assay. The maximum CDA activity of 2.39 ± 0.16 U mg^−1^ was observed with the isolate MS7 (Fig. S2). Hence, the molecular identification of MS7 was undertaken. Homology-based analysis of 16S rRNA has been a gold standard for bacterial identification. The 16S rRNA GenBank BLAST results of the isolate MS7 showed a 99.84% match with *B. aryabhattai* B8W22 (NCBI Reference Sequence: NR_115953.1)*.* The phylogenetic tree was constructed by selecting homologous sequences with *Bacillus niacin* strain as the outgroup (Fig. 1). The isolate MS7 has been submitted for general deposition to National Culture for Microbial Resource (NCMR), India with the accession ID: MCC 3987.

### Gene identification, annotation, and cloning

Neighbourhood-based homology search with *B. megaterium* putative polysaccharide deacetylase (Gene ID: NZ_CP009920.1) gene helped to decode the chitin deacetylase gene from *B. aryabhattai* whole genome (Genome ID: NZ_JYOO01000001.1). The *B. aryabhattai* CDA gene had a 100% query cover with a 97% identity with *B. megaterium* putative polysaccharide deacetylase gene. The identified *B. aryabhattai* chitin deacetylase (*Ba*CDA) gene was annotated in the NCBI gene databank, and the nucleotide sequence data are available in the third-party section of the DDBJ/ENA/GenBank databases with TPA: BK010747 as the accession number.

The virtual clone plasmid was constructed using SnapGene software (Fig. S3). The *Ba*CDA gene (~ 765 bp) from the genomic DNA of *B. aryabhattai* was amplified with *Nde*I and *Xho*I restriction sites incorporated via the primers (Fig. S4). The double restricted (*Nde*I and *Xho*I) amplicon was ligated into the *Nde*I and *Xho*I digested pET-22b (+) vector. The recombinant vector of size ~ 6132 bp was later confirmed by agarose gel electrophoresis. Secondary confirmation was done by amplification with gene-specific primers and double digestion with *Nde*I and *Xho*I restriction enzymes.

*Ba*CDA(pET22b+) was amplified with the T7 promoter and T7 terminator primer for confirmation of cloning. The nucleotide sequence was translated using BioEdit software (Hall et al. 2011). The structure-based sequence alignment showed that the *Ba*CDA had a high sequence similarity in the conserved motifs to the existing CDA of other microorganisms. The protein structure has the (β/α)_8_ barrel topology that is the characteristic of CE-4 category enzyme (Fig. S5). The sequence consists of a catalytic domain (NodB) at C-terminal with a signal peptide of a 23-amino acid length, with Leucine at N-terminus and valine at the C-terminus (data not shown).

### Expression optimization and purification

The cloned vector was transformed into *E. coli* Rosetta pLysS cells. The initial expression experiments were performed in Luria–Bertani (LB) medium with 1 mM Isopropyl β-d-1-thiogalactopyranoside (IPTG) as the inducer. The induced cells at temperatures 37 °C and 16 °C were evaluated for soluble expression. The cells induced at 16 °C showed expression as a soluble intracellular fraction. Other parameters optimized to maximize the expression were, the optical density of the cells at the time of induction, the concentration of IPTG, and the post-induction culture time. Based on these preliminary optimization experiments, the induction OD_600_ was 0.6. The optimum concentration of IPTG was 0.1 mM and the 16 h culture time post induction (Data have not shown). Further expression studies involving IPTG induction were conducted with these optimized conditions.

In addition to the IPTG induction, lactose induction (as auto-inducer) was also tested for the *Ba*CDA expression. Induction conditions with IPTG and lactose were compared in three reported media viz. Luria Bertani (LB), Yeast extract-tryptone broth (2YT), and Terrific Broth (TB). The expression and the growth of the culture were determined at an interval of 24 h and 48 h. After incubation of 24 h, the *Ba*CDA expression (protein corresponding to 29 kDa) was observed in all the above three media with IPTG as the inducer. In the lactose auto-induction media, no expression was observed at the 24th h (Fig. S6A). The maximum expression at the 24th h time point was observed in the LB media. Upon extending the culture duration to 48 h, maximum expression was observed in TB lactose auto-induction media (Fig. S6B). Therefore, TB lactose auto-induction medium was used for further experiments.

Using 5 g of induced cell pellet, 25 mL of cell lysate was prepared and diluted in 25 mL of lysis buffer to give a total protein of 275 mg and specific activity of 14.57 U mg^−1^ (Table 1). The recombinant *Ba*CDA was purified to homogeneity and the apparent molecular weight of the purified *Ba*CDA was 29 kDa (Fig. S6C). The purified *Ba*CDA elutions were pooled, dialyzed, and concentrated. After concentrating, the total recovered protein was 23.10 mg and the specific activity of purified *Ba*CDA was 38.89 U mg^−1^. The activity yield was 22.42% and the purification fold was 2.67 (Table 1).

**Table 1:** The specific activity and yield of *Ba*CDA

Sample	Total protein (mg)	Total activity (U)	Specific activity (U mg^−1^)	Purification fold	Activity yield (%)
Culture lysate	275.00	4006.75	14.57	1.00	100.00
Purified *Ba*CDA	23.10	898.36	38.89	2.67	22.42

The enzyme activity and concentration were determined by acetate assay and Bradford’s assay, respectively. One unit of the enzyme is defined as the activity which released 1 μmol of acetate from the substrate per mg of enzyme per minute. The values correspond to the average and standard deviation of experiments done in triplicates

### Expression scale-up and investigation of lactose induction

**Fig. 2:**
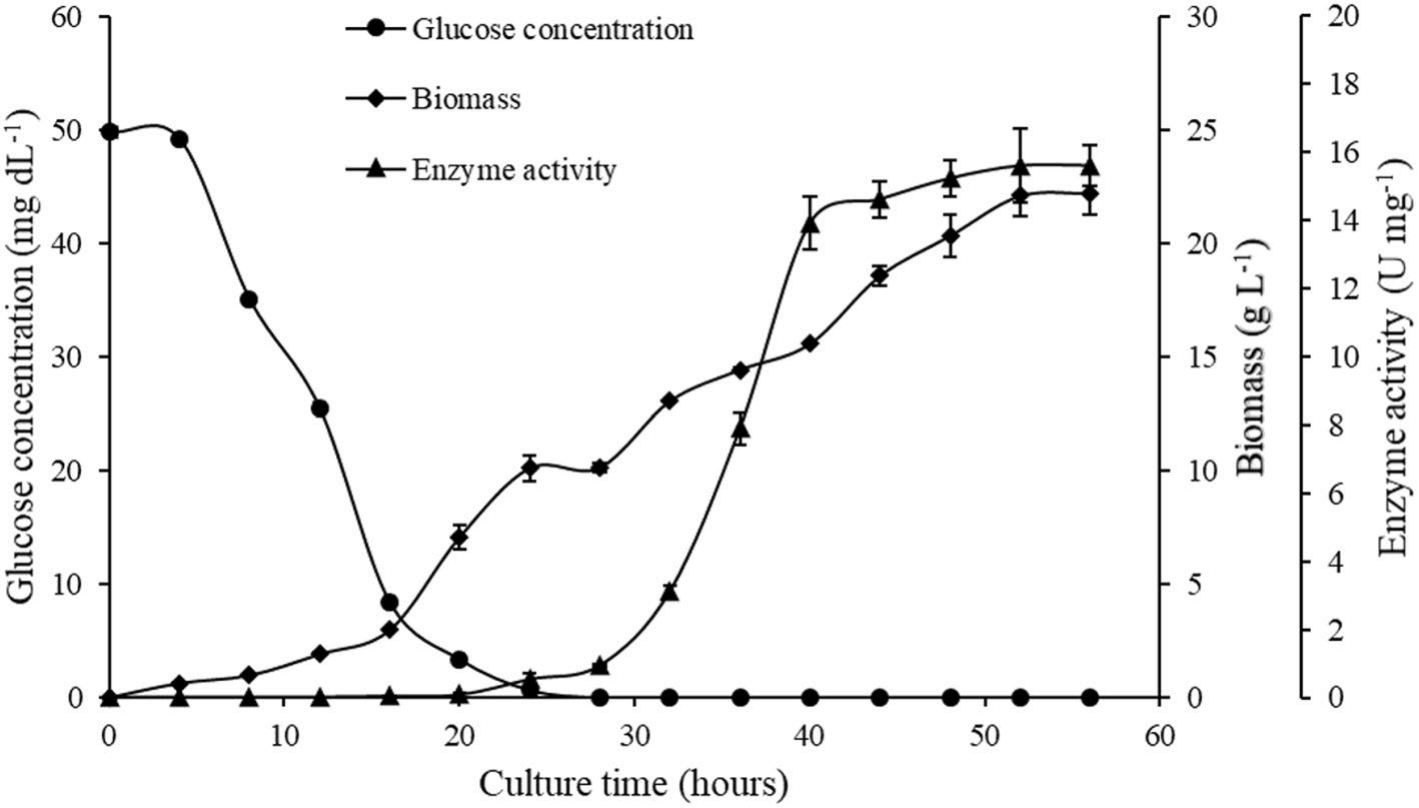
Expression study in TB lactose-induced media. Glucose concentration (●), cell biomass (♦), and enzyme activity (▲) plotted against time (*X*-axis). All experiments were performed in triplicates and error bars represent the standard deviation of the mean

Based on expression studies, the expression was scaled up to 1 L in a shake flask under optimized conditions. Meanwhile, to demonstrate the lactose induction, the biomass of the culture, glucose concentration, and enzyme activity were estimated. The culture had an initial lag phase of 16 h followed by the first exponential phase lasting for the next 8 h. This was followed by flattening of the curve and then the second exponential phase after 28 h (Fig. 2). The growth reached the final stationary phase after 56 h with biomass of 22.26 ± 0.98 g L^−1^ (Fig. 2). The initial concentration of the glucose at 0 h was 50 mg dL^−1^ which declined gradually to reach zero after 24 h. The second log phase was observed after the glucose was exhausted at 24 h (Fig. 2). The *Ba*CDA expression starts after 28 h, as *E. coli* cells start lactose consumption and allolactose was produced to induce the expression. The enzyme activity in the culture lysate was observed after 28 h and reaches 14.05 ± 0.51 U mg^−1^ within 44 h (Fig. 2). The maximum biomass was at 56th h and the enzyme activity at this point was found to be 15.01 ± 0.58 U mg^−1^. Therefore, 56 h of culture was used for further expression studies.

### *Ba*CDA characterization

**Fig. 3:**
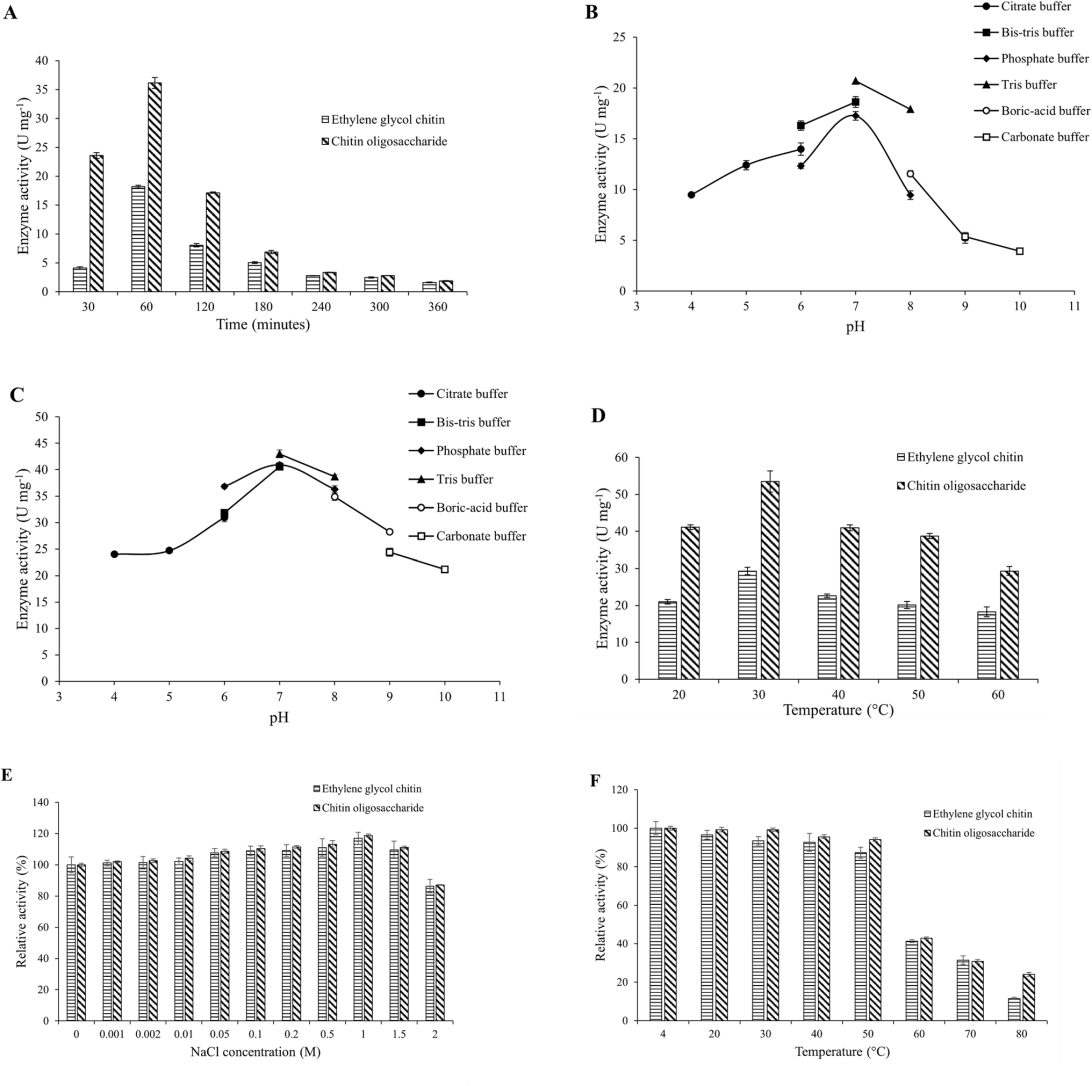
**A** Recombinant *Ba*CDA enzyme velocity study. **B** Recombinant *Ba*CDA activity in buffer system covering the pH range 4–10, when EGC was used as substrate. (Citrate buffer—●, Bis–tris buffer—■, Phosphate buffer—♦, Tris–HCl buffer—▲, Boric acid buffer—○, Carbonate buffer—□). **C** Recombinant *Ba*CDA activity in buffer system covering the pH range 4–10, when COS was used as substrate. (Citrate buffer—●, Bis–tris buffer—■, Phosphate buffer—♦, Tris–HCl buffer—▲, Boric acid buffer—○, Carbonate buffer—□). **D** The optimum temperature for *Ba*CDA, the enzyme activity was determined at the temperature ranging from 20 to 60 ºC. **E** The salt tolerance of *Ba*CDA, the enzyme activity was investigated in presence of NaCl concentration ranging from 0.001 to 2 M. **F** The thermo-stability of *Ba*CDA, the enzyme activity was tested by incubating the enzyme at a different temperature ranging from 4 to 80 °C for 24 h. All experiments were performed in triplicates and error bars represent the standard deviation of the mean

The enzyme velocity of the purified *Ba*CDA was estimated between 30 and 360 min using EGC and COS as substrates (Fig. 3A). Maximum enzyme activities of 18.21 ± 0.25 U mg^−1^ and 36.13 ± 0.94 U mg^−1^ were observed at 60 min with EGC and COS, respectively. After 60 min, the enzyme activity gradually decreased and reached 1.61 ± 0.07 U mg^−1^ and 1.86 ± 0.06 U mg^−1^ with EGC and COS, respectively (Fig. 3A).

Therefore, further experiments were carried out for one hour. The pH optima of *Ba*CDA were studied in the pH ranges of 4–10. The *Ba*CDA showed maximum activity at pH 7 in all buffer conditions in both substrates. The enzyme activity was maximum 20.69 ± 0.20 U mg^−1^ and 42.99 ± 0.71 U mg^−1^ with substrate EGC and COS, respectively, in of 50 mM Tris–HCl buffer at pH 7 (Fig. 3B and C). An increase or decrease in the pH results in the loss of enzyme activity in all buffer conditions. Therefore, the optimum buffer and pH condition were set to 50 mM Tris–HCl and pH 7, respectively.

The temperature optima were studied in ranges between 20 and 60 ºC. The maximum enzyme activity was found to be at 30 ºC. The maximum enzyme activity was 29.28 ± 1.00 U mg^−1^ and 53.47 ± 2.90 U mg^−1^ with substrate EGC and COS, respectively, at 30 ºC (Fig. 3D). Further, an increase in the reaction temperature results in activity loss to 18.26 ± 1.32 U mg^−1^ and 29.32 ± 1.19 U mg^−1^ with substrate EGC and COS, respectively, at 60 ºC (Fig. 3D).

CDA is known to be metalloenzymes; hence, the effect of the metal co-factors was studied. The effect of metal ions at a concentration of 1 mM on the enzyme activity was assayed. The presence of metal chelators like EDTA was also analysed (Table 2). The inclusion of 1 mM metal ions, such as K^+^, Na^+^, Ca^2+^, Co^2+^, Mn^2+^, Mg^2+^, Fe^2+^, Ni^2+^, and Zn^2+^, resulted in 89.36 ± 4.45%, 97.95 ± 7.82%, 91.75 ± 3.96%, 84.67 ± 4.99%, 83.17 ± 3.30%, 132.93 ± 7.26%, 62.51 ± 6.03%, 58.87 ± 8.28%, and 68.57 ± 8.36% of the original activity, respectively, when EGC was used as substrate (Table 2). While with the substrate COS, the 1 mM metal ion K^+^, Na^+^, Ca^2+^, Co^2+^, Mn^2+^, Mg^2+^, Fe^2+^, Ni^2+^, and Zn^2+^ resulted in 84.94 ± 5.94%, 98.24 ± 5.23%, 93.98 ± 6.00%, 87.53 ± 3.54%, 85.22 ± 2.90%, 135.34 ± 3.26%, 69.04 ± 5.82%, 54.74 ± 8.37%, and 67.18 ± 5.02% of the original activity, respectively. When 1 mM EDTA was added to the enzymatic reaction, 40.83 ± 5.71% and 48.83 ± 2.70% of the original enzymatic activity were retained with the substrate EGC and COS respectively (Table 2). Acetate is one of the by-products of the chitin deacetylase reaction and caused feedback inhibition. Hence, the inhibitory action of acetate ion on the enzyme activity was studied at a concentration range of 0.1 to 10 mM. An increasing trend of inhibition was observed with an increase of acetate from 0.1 to 10 mM. The maximum inhibition was 49.88 ± 3.89% and 46.95 ± 2.43% in presence of 10 mM of acetate was observed with the substrate EGC and COS respectively (Table 2). A titration of the Mg^2+^ was made in the standard assay conditions. An increase of Mg^2+^ concentration from 0.5 to 1.5 mM resulted in a 137.43 ± 4.74% and 139.22 ± 3.99% increased activity with EGC and COS, respectively. This further incremented to 142.43 ± 7.13% and 146.88 ± 4.09% with EGC and COS, respectively, in presence of 2 mM Mg^2+^. Further increase of Mg^2+^ concentration resulted in a decreased activity (Table 2). Hence, 2 mM Mg^2+^ was used for further experiments.

**Table 2:** Effect of metal co-factors on *Ba*CDA enzyme activity

Metal ion	Concentration	Relative activity (%)	
		Ethylene glycol chitin	Chitin oligosaccharide
Control	–	100 ± 11.23	100.00 ± 5.82
K^+^	1 mM	89.36 ± 4.45	84.94 ± 5.94
Na^+^	1 mM	97.95 ± 7.82	98.24 ± 5.23
Ca^2+^	1 mM	91.75 ± 3.96	93.98 ± 6.00
Co^2+^	1 mM	84.67 ± 4.99	87.53 ± 3.54
Mn^2+^	1 mM	83.17 ± 3.30	85.22 ± 2.90
Fe^2+^	1 mM	62.51 ± 6.03	69.04 ± 5.82
Ni^2+^	1 mM	58.87 ± 8.28	54.74 ± 8.37
Zn^2+^	1 mM	68.57 ± 8.36	67.18 ± 5.02
EDTA	1 mM	40.83 ± 5.71	48.83 ± 2.70
Acetate	0.1 mM	96.75 ± 7.84	94.34 ± 5.01
	1 mM	92.77 ± 3.32	82.16 ± 1.40
	10 mM	49.88 ± 3.89	46.95 ± 2.43
Mg^2+^	0.5 mM	110.49 ± 8.81	125.16 ± 3.64
	1 mM	132.93 ± 7.26	135.34 ± 3.26
	1.5 mM	137.43 ± 4.74	139.22 ± 3.99
	2 mM	142.43 ± 7.13	146.88 ± 4.09
	2.5 mM	141.32 ± 7.40	143.85 ± 3.32
	3 mM	141.50 ± 7.95	143.64 ± 0.19

The effect of metal ions was investigated taking a reaction without any metal ion as control. The values correspond to the average and standard deviation of experiments done in triplicates

In the study, to evaluate the salt tolerance of *Ba*CDA, the enzyme activity without NaCl was considered as 100%. The relative enzyme activity increased with an increment in NaCl concentration and was found maximum in presence of 1 M NaCl. The relative activity detected with 1 M NaCl was 116.98 ± 3.87% and 118.70 ± 0.98% for EGC and COS, respectively (Fig. 3E). Further increase in the salt concentration to 2 M led to a decrease in the activity (86.27 ± 4.33% and 87.08 ± 0.21% for EGC and COS, respectively). The thermo-stability of the enzyme was determined by incubating the enzyme at a different temperature ranging from 4 to 80 °C in presence of 2 mM Mg^2+^ and 1 M NaCl for 24 h at a steady state. The enzyme stored at 4 °C was considered as 100% enzyme activity. The enzyme was stable up to 50 °C with less significant loss of activity (87.27 ± 2.85% and 94.08 ± 0.92% for EGC and COS, respectively). However, the enzyme activity decreased significantly on increasing the temperature to 60 °C. The relative enzyme activity on incubating at 60 °C was 41.35 ± 0.66% and 42.86 ± 0.60% with EGC and COS, respectively (Fig. 3F).

### Kinetic parameter studies of *Ba*CDA

**Fig. 4:**
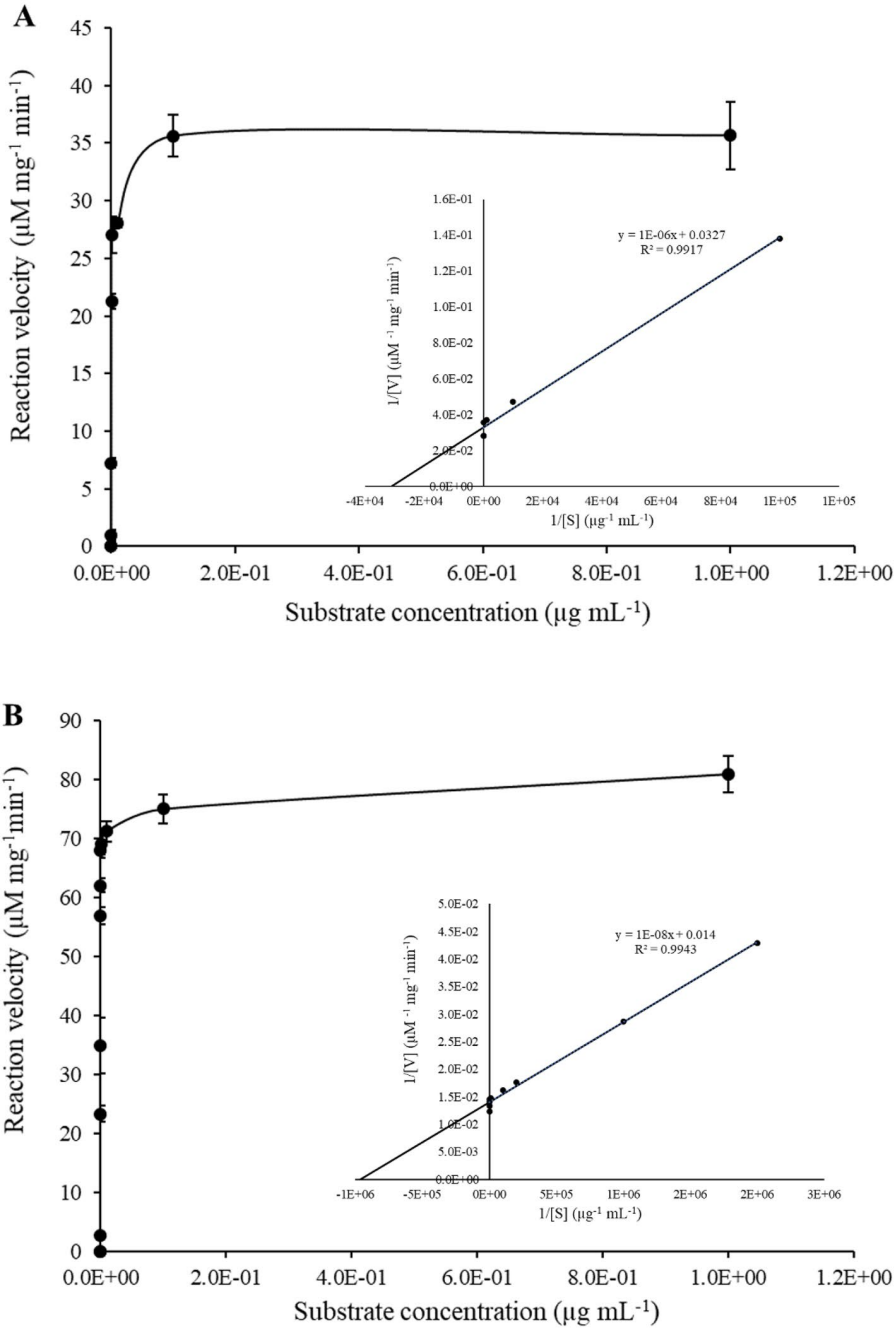
**A** Michaelis–Menten kinetic curve generated using EGC as a substrate. **B** Michaelis–Menten kinetic curve generated using COS as a substrate. All experiments were performed in triplicates and error bars represent the standard deviation of the mean

The kinetic parameters of recombinant *Ba*CDA were performed with EGC at concentrations from 1.00 to 5.00E−7 µg mL^−1^ and estimated with acetate released during the reaction. The kinetics of the enzyme showed an excellent fit to the Michaelis–Menten equation (Fig. 4A). The *K*_m_ value of *Ba*CDA was 3.06E−05 µg mL^−1^, and the maximal velocity was 3.06E + 01 µM mg^−1^ min^−1^. The turnover number and the catalytic efficiency value were 3.27E + 04 s^−1^ and 1.07E + 09 µg^−1^ mL^−1^ s^−1^, respectively, according to the *K*_m_ and *V*_max_ (Table 3).

**Table 3:** Kinetic parameters of Chitin deacetylase from *B. aryabhattai*, EGC, and COS were taken as a substrate

Substrate	*K*_m_ (µg mL^−1^)	*V*_max_ (µM mg^−1^ min^−1^)	*K*_cat_ (s^−1^)	*K*_cat_/*K*_m_ (µg^−1^ mL^−1^ s^−1^)
Ethylene glycol chitin	3.06E−05	3.06E+01	3.27E+04	1.07E+09
Chitin oligosaccharides	7.14E−07	7.14E+01	1.40E+06	1.96E+12

The values correspond to the average and standard deviation of experiments done in triplicates

The kinetic parameters of recombinant *Ba*CDA were performed with COS at concentrations from 1.00 to 1.00E−8 µg mL^−1^ and estimated with acetate released during the reaction. The kinetics of the enzyme showed an excellent fit to the Michaelis–Menten equation (Fig. 4B). The *K*_m_ value of *Ba*CDA was 7.14E−07 µg mL^−1^, and the maximal velocity was 7.14E + 01 µM mg^−1^ min^−1^. The turnover number and the catalytic efficiency value were 1.40E + 06 s^−1^ and 1.96E + 12 µg^−1^ mL^−1^ s^−1^, respectively, according to the *K*_m_ and *V*_max_ (Table 3).

## Discussion

Seafood waste processing generates chitin, an undervalued waste. Despite being a second-abundant biopolymer, the applications are limited due to their crystalline nature. This limitation can be addressed by converting chitin to chitosan (Yadav et al. 2019). To date, the commercial production of chitosan is carried out through chemical deacetylation which results in a random pattern of deacetylation and the chemicals used for this conversion are hazardous to the environment (Santos et al. 2020). The greener route for the conversion is using enzymes like CDA. As marine is the major source for chitin, it also upholds the enzymes converting it to chitosan (Younes and Rinaudo 2015). The reports on CDA-producing bacteria are sparse. *Vibrio species* and *Nitratireductor aquimarinus* are the only species reported from the marine environment. Thus, the marine bacterial ecosystem as a source of CDA can be widely explored (Ghormade et al. 2010).

In the present study, we have isolated 15 bacteria from 40 m sea sediment with 12° 48′ N and 74° 40′ E as the coordinates. The isolates were screened on a receptor-based screening plate and out of fifteen, four isolates were having CDA activity (Pawaskar et al. 2019). The reason for the growth of other organisms on the colloidal chitin plate could be due to the production of other chitinolytic enzymes (Patel et al. 2007; Kaczmarek et al. 2019; Schmitz et al. 2019; Mathew et al. 2021). The CDA production of all the four isolates was quantitated by the spectrophotometric-based method and isolate MS7 yielded the maximum CDA activity. The 16S rRNA gene of the isolate MS7 had a 99% match with *B. aryabhattai* B8W22. The first mention of *B.aryabhattai* was in the report by Shivaji et al*.* where they had isolated the strain from atmospheric air using cryotubes at an altitude of 27–30 km (Shivaji et al. 2009). The second mention of *B. aryabhattai* was in the work of Semanti Ray et al. where they report the extra-terrestrial microorganism to be found in the Indian Sub-continent, evidence in support of the Theory of Panspermia. In continuation of proving the Theory of Panspermia, we report the isolation of *B. aryabhattai* from Arabian Sea sediment at a depth of 40 m depth. The microorganisms were isolated under high salt conditions by providing synthetic seawater and thus, all the isolates were halophilic (Dalmaso et al. 2015). The CDA gene in the *B. aryabhattai* genome was identified by a homology-based method using the putative CDA gene from *B. megaterium*. The identified *Ba*CDA gene was annotated in the NCBI gene databank, available in the third-party section of the DDBJ/ENA/GenBank databases under the accession number TPA: BK010747 for the *Ba*CDA gene. The annotated *Ba*CDA gene (~ 765 bp) was cloned in the pET-22b (+) vector and overexpressed in *E. coli* Rosetta pLysS cells to overexpress a 29 kDa enzyme. The molecular weight of CDA as reported from various sources range between 25 and 80 kDa (Ghormade et al. 2010; Zhao et al. 2010). The temperature of induction was an important criterion, and the optimization experiments were conducted at two temperatures viz 37 °C and 16 °C. At 37 °C, the expression of CDA as inclusion bodies. This has been supported by a similar finding wherein lower temperatures after induction help in the proper folding of the protein in *E. coli* (Rosano and Ceccarelli 2014). Hence, further expression studies were performed at 16 °C.

In the T7 lac promoter vector systems, IPTG can be replaced by lactose as an inducer. The inclusion of lactose not only reduces the overall cost of the process, but also reduces the toxicity caused to the host (Blommel et al. 2007). Hence, in the present study, the inducer optimization was also performed based on literature reports. The media with lactose were designated as auto-induction (AI) media. The induction in AI media was controlled with the inclusion of glucose as a component. To obtain the maximum expression level with IPTG induction and lactose induction, the three commonly used media viz. LB, 2YT, and TB media were used. The expression and the cell density were highest in TB lactose induction media as compared to the other combinations of media studied. This could be due to the inclusion of glycerol in the media. Glycerol when coupled with glucose and lactose in the culture media was known to positively impact recombinant protein production (Blommel et al. 2007). The TB lactose induction medium was optimized for *Ba*CDA expression, and the obtained protein was purified by Ni–NTA affinity chromatography. We could obtain the purified *Ba*CDA to homogeneity with the single-step purification protocol. The purified *Ba*CDA had an estimated molecular mass of 29 kDa as observed in the 12.5% SDS-PAGE. The *Ba*CDA expression was scaled up to 1 L TB lactose induction media. Catabolite repression was observed in the presence of glucose as it was readily metabolized by the cell and thus prevented the uptake of lactose. Similar findings have also been observed by other authors (Kopp et al. 2018). The availability of glucose was exhausted after 24 h; this led to the uptake of lactose by the cells. Lactose upon conversion to allo-lactose led to the induction of the T7 lac promoter present in the pET-22b (+) vector. Hence, the *Ba*CDA expression upregulated after the 28th h of growth. Glycerol present in the TB media does not result in catabolite repression; hence, higher expression yields were obtained in TB lactose induction media as compared to LB and 2YT lactose induction media. This is in agreement with the findings by other authors (Studier 2005; Blommel et al. 2007; Kopp et al. 2018).

To date, reported bacterial CDAs were active on either chitin polymer or chitin oligosaccharides. Only a few fungal CDAs are reported for activity on substrates with a wide range of DP (Kaczmarek et al. 2019). The purified *Ba*CDA reported in this study was active on both EGC and COS. Therefore, characterization of *Ba*CDA was done using ethylene glycol chitin as well as chitin oligosaccharides. Before initiating the pH and temperature characterization of *Ba*CDA, we investigated the initial velocity required by the enzyme to convert EGC and COS. We found that the conversion rate was faster in the initial phase leading to a decrease in the peak after 60 min of incubation. A similar finding was also observed by Win and Stevens with fungal *Absidia coerulea* CDA (Win and Stevens 2001). This decrease could be due to the build-up of acetate as the by-product of the deacetylation reaction which has been reported to result in feedback inhibition (Zhao et al. 2010). A similar inhibitory effect of acetate on CDA has also been reported in *Absidia orchidis vel coerulea*, *Amylomyces rouxii*, *Lichtheimia corymbifera*, *Penicillium oxalicum*, *Rhizomucor miehei*, and *Saccharomyces cerevisiae* (Ghormade et al. 2010)*.* In the present study, *Ba*CDA enzyme activity was found to be maximum with a 50 mM Tris–HCl buffer at a pH of 7, and the optimum temperature was found to be 30 ºC. This was in agreement with the pH ranges found in the microbial CDA where groups have reported the pH optima ranging from 4.5 to 12 (Grifoll-Romero et al. 2018). *Ba*CDA displayed a wide range of pH tolerance with effective conversion in both the acidic and the alkaline range with the maxima at pH 7. *Nitratireductor aquimarinus*, the recently characterized marine bacteria has the pH optima at 8 and temperature maxima at 30 ºC (Chai et al. 2020). CDA is known to be metalloenzymes and thus becomes activated with divalent metal co-factors. In the present study, divalent ion Mg^2+^ at the concentration of 2 mM led to an enhancement in the enzyme activity. For most of the CDA reported, Co^2+^ acts as an activator/enhancer (Ghormade et al. 2010). Mg^2+^ as a co-factor has been reported in CDA activity obtained from *B. amyloliquefaciens*, *Lichtheimia corymbifera*, *Penicillium oxalicum*, and *Rhizopus circinans* organisms (Grifoll-Romero et al. 2018). On the other hand, Sr^2+^ was the activator in CDA reported in *Nitratireductor aquimarinus* (Chai et al. 2020). In the present study, EDTA with a concentration of 1 mM inhibited the enzyme activity to 40.83 ± 5.71% and 48.83 ± 2.70% when EGC and COS were used as substrate, respectively, which was higher than its marine counterpart *Nitratireductor aquimarinus*, which reported a loss of 70% enzymatic activity (Chai et al. 2020). The enzyme physio-chemical properties are mainly due to the source of the enzyme. As the *BaCDA* gene was amplified from *B. aryabhattai* B8W22, a marine isolate, it was expected to be halotolerant and thermostable (Jin et al. 2019). The enzyme displayed an improved activity with the inclusion of 1 M NaCl in the assay solution. The *Ba*CDA was thermostable up to 50 °C for 24 h. Above 50 °C, *Ba*CDA enzyme activity decreases significantly on incubating for 24 h. In recent years, use for extremozyme showed potential for industrial application due to the ease of biotechnological processes (Dumorné et al. 2017). There are several studies on extremozymes for their industrial application with few chitinolytic enzymes too reported (Niehaus et al. 1999; Wang et al. 2016; Paranetharan et al. 2018). There are reports on thermostable chitin deacetylase (Grifoll-Romero et al. 2018). However, no reports on exploring the salt-tolerant CDA were found. The halotolerant and thermo-stability property of *Ba*CDA makes it novel with high industrial applicability.

The kinetic parameters for *Ba*CDA on EGC and COS were obtained from the Lineweaver–Burk plot analysis and the enzyme reaction rates seemed to follow the Michaelis–Menten kinetics. The kinetic parameters of *Ba*CDA can be compared with other reported CDAs. The CDA from *B. amyloliquefaciens* having the *K*_m_ of 9.96E−06 µg mL^−1^, *V*_max_ of 4.78E + 06 µM min^−1^, and *K*_cat_ of 5.18E + 03 s^−1^ under optimum conditions (Bhat et al. 2019). The kinetic parameters with the fungal CDA from *Colletotrichum lindemuthianum* on EGC have reported the *K*_m_, *V*_max_ and *K*_cat_ of 2.55 mM, 51.3 µM mg^−1^ min^−1^ and 27.1 s^−1^, respectively (Tokuyasu et al. 1996). The other fungal CDA studied on EGC was *Aspergillus nidulans* where *K*_m_ of 4.92E + 03 µg mL^−1^, *V*_max_ was 0.77 µM mg^−1^ min^−1^ and *K*_cat_ of 6.25 s^−1^ have been reported (Wang et al. 2010). These results help us to conclude that the *Ba*CDA has a more affinity than that of fungal CDA but less affinity than that of recombinant CDA from *B. amyloliquefaciens* when EGC was used as substrate. To date, only fungal CDAs are reported for activity towards COS. *Ba*CDA is the first bacterial recombinant CDA reporting for activity towards EGC as well as COS.

In conclusion, the marine environment can be explored to find novel extremophilic microorganisms producing extremozyme CDAs. The products obtained after enzymatic modification yield specific deacetylation fingerprints. This makes them a better choice for biomedical applications (Morin-Crini et al. 2019). The use of extremozyme leads to reduce the operational cost, risk of contamination during the process, and due to the stability, high productivity is also obtained (Dumorné et al. 2017; Jin et al. 2019). Thus, *Ba*CDA being the extremozyme, could be used for the industrial production of enzymatically derived chitosan polymer as well as chitosan oligosaccharides with the known pattern of deacetylation. Even though the K_m_ and V_max_ values showed good affinity towards the substrates EGC as well as COS, it was observed that the *Ba*CDA had more affinity towards COS because of the short length of the substrate. Therefore, *Ba*CDA could be used as chitin oligosaccharide deacetylase as well as chitin deacetylase.

## Supplementary Information

Below is the link to the electronic supplementary material.

Fig. S1 Screening of CDA-producing organism on colloidal chitin receptor plate. Spot inoculation of 15 isolates was done and observed at an interval of 24 h for 4 days. The isolates MS7, MS11, MS12, and MS13 showed fluorescence around the colony due to CDA activity. Fig. S2 Crude enzyme activity of four positively screened isolates by acetate assay kit. All experiments were performed in triplicates and error bars represent the standard error of the mean. Fig. S3 Plasmid construct of BaCDA in pET22b (+) vector. Fig. S4 Agarose gel electrophoresis of the BaCDA gene amplified from the gDNA of B. aryabhattai. The size of the amplicon was ~765 bp. Fig. S5 Multiple sequence alignment of BaCDA with other known CE-4 category enzyme sequences. The BaCDA amino acid sequence aligned with Bacillus subtilis PdaA [PDB ID: 1W17], Streptococcus pneumoniae SpPgdA [PDB ID: 2C1G], Streptomyces lividans Pda [PDB ID: 2CC0], Colletotrichum lindemuthianum CDA [PDB ID: 2IW0], Aspergillus nidulans CDA [PDB ID: 2Y8U]. BaCDA has a high sequence similarity in the conserved motifs. The protein structure has the (β/α)8 barrel topology that is the characteristic for CE-4 category enzyme. Fig. S6 12.5% SDS-PAGE of overexpression of BaCDA in E. coli Rosetta pLysS cells in LB, 2YT, and TB media containing IPTG and lactose for induction. (A) After 24 h (B) After 48 h (C) 12.5% SDS-PAGE of BaCDA purification using Ni-NTA affinity chromatography. Lane 1: cell lysate; Lane 2: column flow-through; Lane 3: column wash; Lane 4-7: 250 mM imidazole elusion; Lane 8-10: 500 mM imidazole elusion. M: protein marker (Range: 14 to 97.4 kDa) was run along with the sample (DOCX 2344 KB)
