# Supplementary figures and images for: Cloning, expression, purification and characterization of chitin deacetylase extremozyme from halophilic Bacillus aryabhattai B8W22
Source: 3 Biotech. 2021 Dec 1;11(12):515. doi: 10.1007/s13205-021-03073-3 (PMC8636556; doi:10.1007/s13205-021-03073-3)

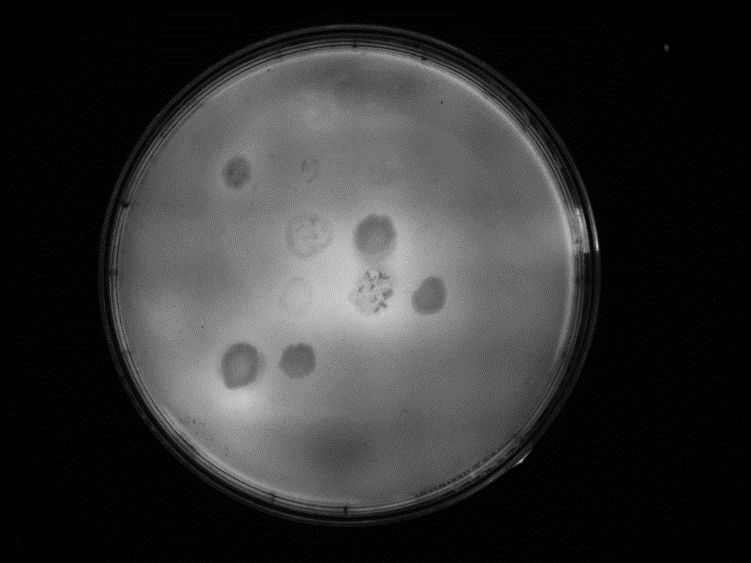


**Fig. S1.**

**
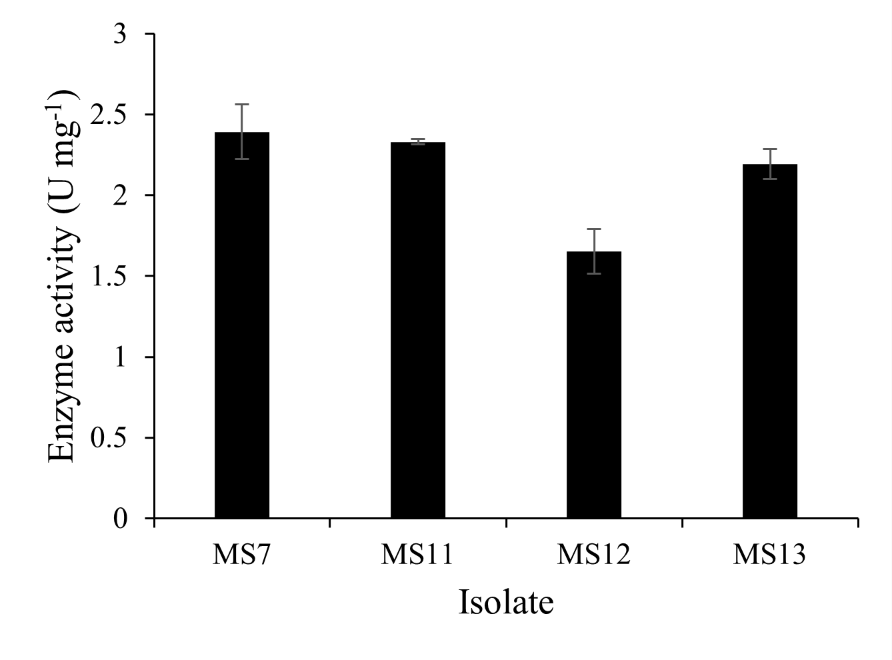
**

**Fig. S2.**

**
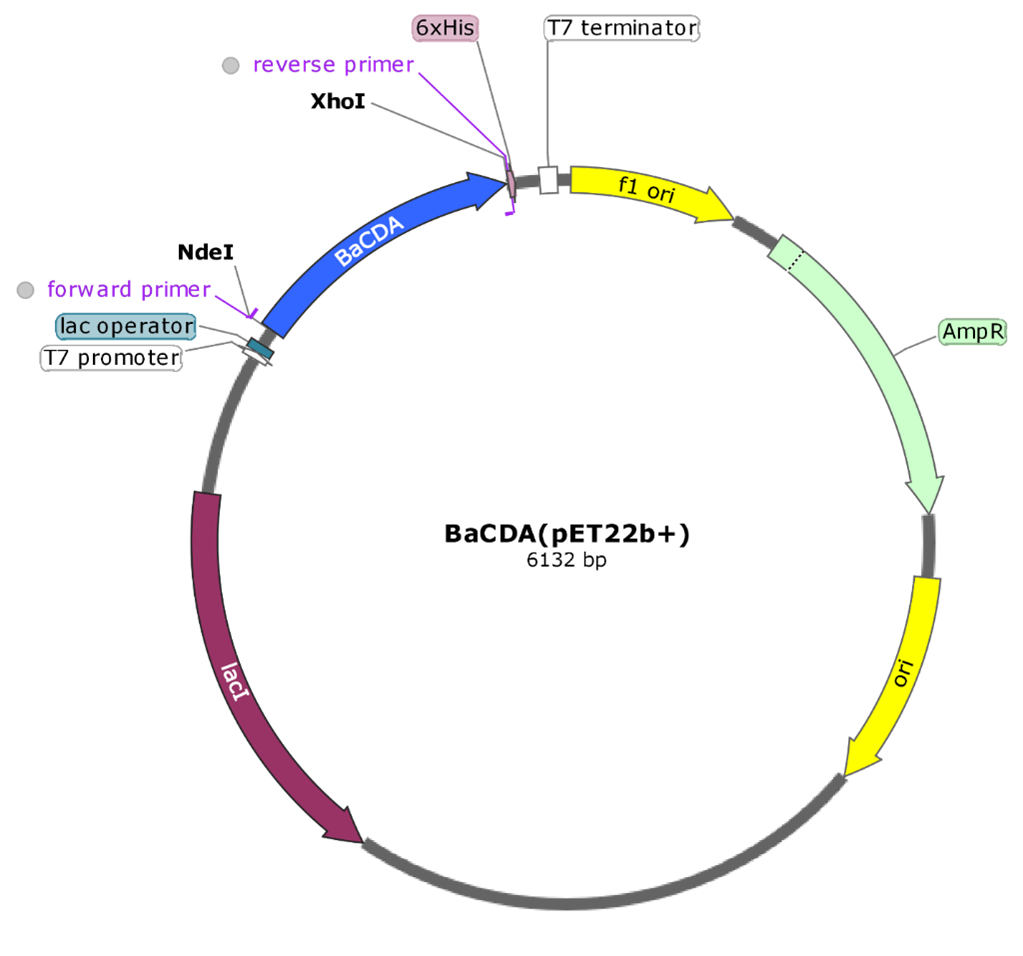
**

**Fig. S3.**

**
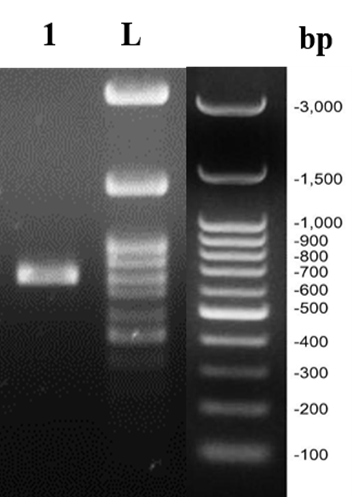
**

**Fig. S4.**

**
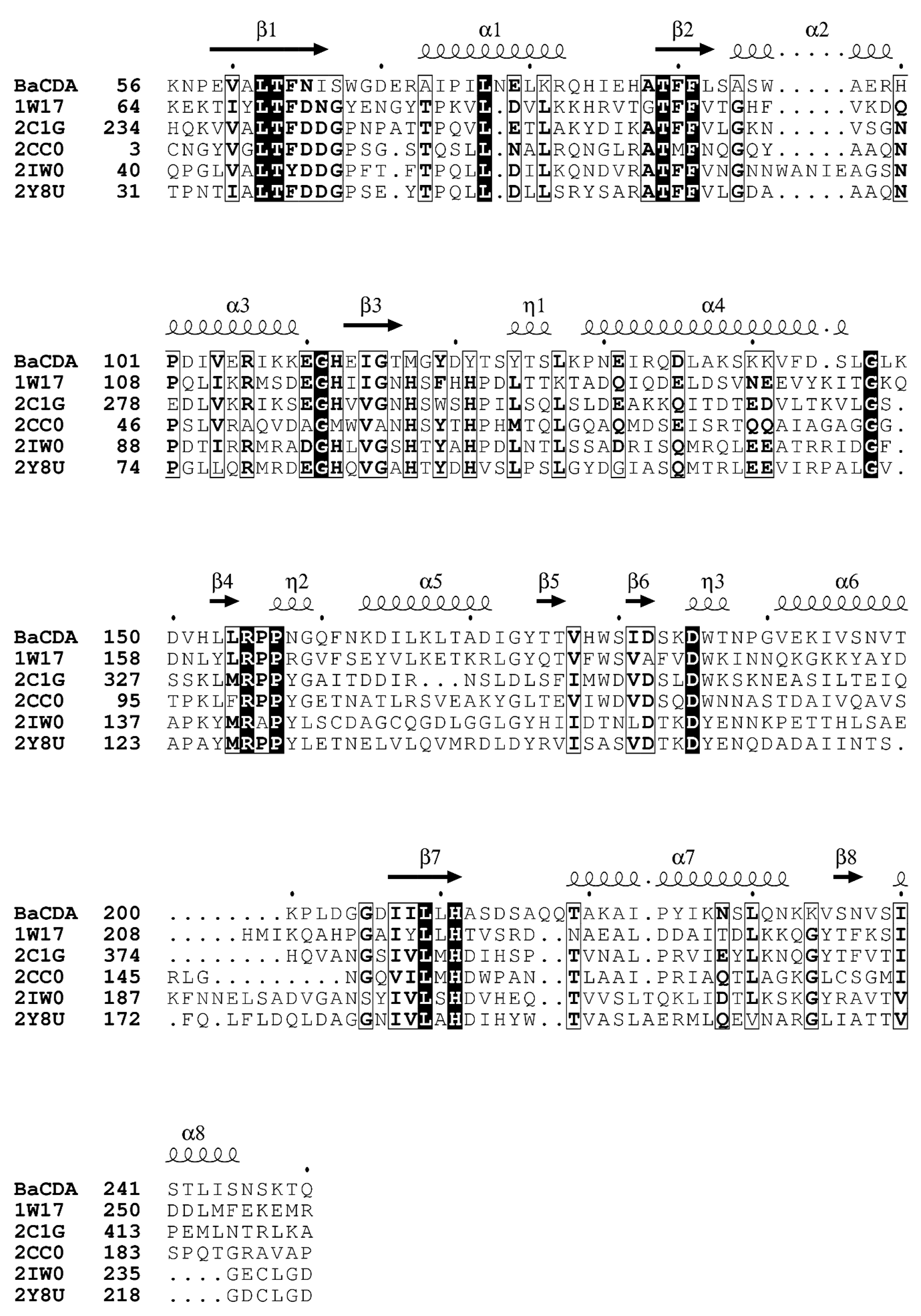
**

**Fig. S5.**

**
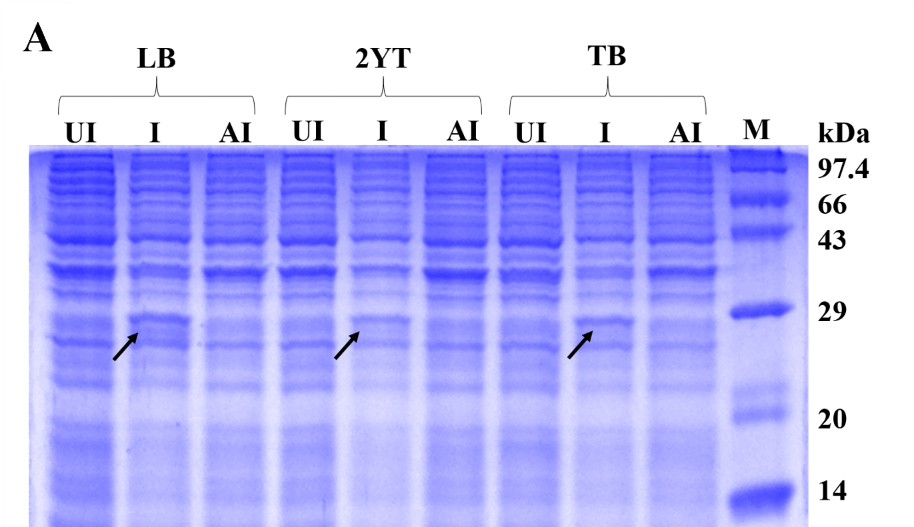

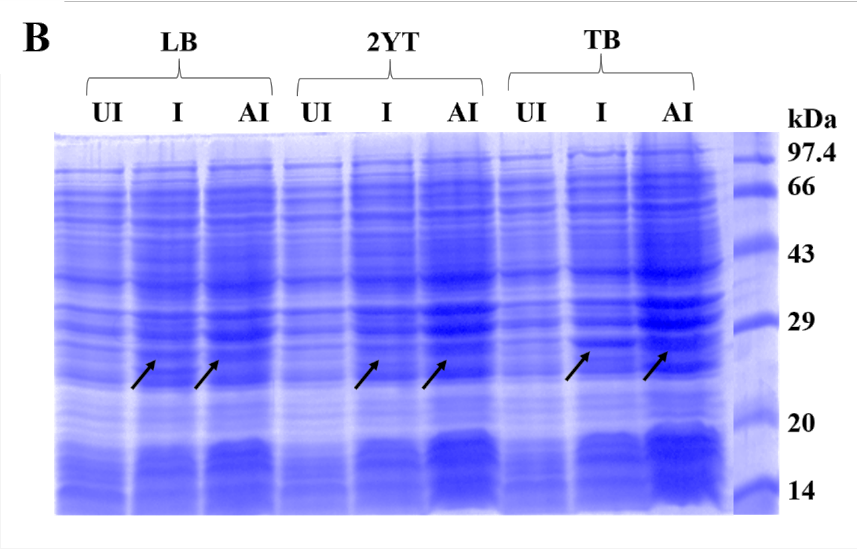

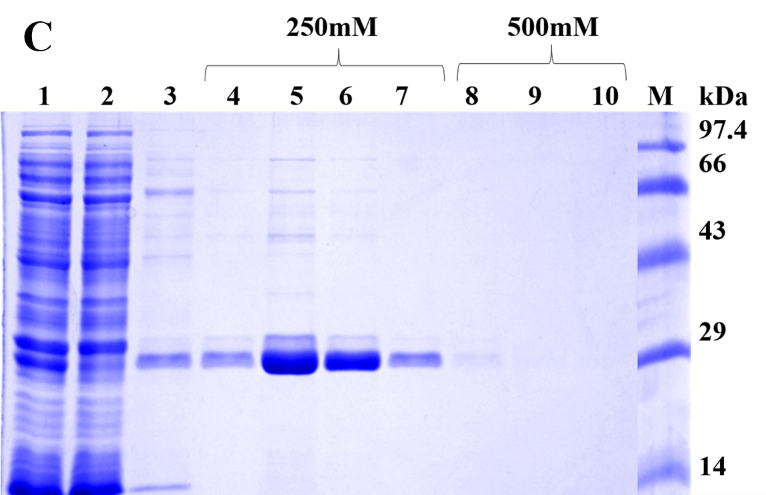
**

**Fig. S6.**

Supplement: Supplementary file 1 — Fig. S1 Screening of CDA-producing organism on colloidal chitin receptor plate. Spot inoculation of 15 isolates was done and observed at an interval of 24 h for 4 days. The isolates MS7, MS11, MS12, and MS13 showed fluorescence around the colony due to CDA activity. Fig. S2 Crude enzyme activity of four positively screened isolates by acetate assay kit. All experiments were performed in triplicates and error bars represent the standard error of the mean. Fig. S3 Plasmid construct of BaCDA in pET22b (+) vector. Fig. S4 Agarose gel electrophoresis of the BaCDA gene amplified from the gDNA of B. aryabhattai. The size of the amplicon was ~765 bp. Fig. S5 Multiple sequence alignment of BaCDA with other known CE-4 category enzyme sequences. The BaCDA amino acid sequence aligned with Bacillus subtilis PdaA [PDB ID: 1W17], Streptococcus pneumoniae SpPgdA [PDB ID: 2C1G], Streptomyces lividans Pda [PDB ID: 2CC0], Colletotrichum lindemuthianum CDA [PDB ID: 2IW0], Aspergillus nidulans CDA [PDB ID: 2Y8U]. BaCDA has a high sequence similarity in the conserved motifs. The protein structure has the (β/α)8 barrel topology that is the characteristic for CE-4 category enzyme. Fig. S6 12.5% SDS-PAGE of overexpression of BaCDA in E. coli Rosetta pLysS cells in LB, 2YT, and TB media containing IPTG and lactose for induction. (A) After 24 h (B) After 48 h (C) 12.5% SDS-PAGE of BaCDA purification using Ni-NTA affinity chromatography. Lane 1: cell lysate; Lane 2: column flow-through; Lane 3: column wash; Lane 4-7: 250 mM imidazole elusion; Lane 8-10: 500 mM imidazole elusion. M: protein marker (Range: 14 to 97.4 kDa) was run along with the sample (DOCX 2344 KB) [file 13205_2021_3073_MOESM1_ESM.docx]
